# Perceived risks, reasons for use, and barriers to cessation among youth who use multiple tobacco products

**DOI:** 10.1371/journal.pone.0335019

**Published:** 2025-11-07

**Authors:** Sierra L. Patterson, Isabelle Duguid, Sonia A. Clark, Seth M. Noar, Allison J. Lazard, James F. Thrasher, Adam O. Goldstein, Sarah D. Kowitt

**Affiliations:** 1 Department of Epidemiology, Gillings School of Global Public Health, University of North Carolina at Chapel Hill, Chapel Hill, North Carolina, United States of America; 2 Department of Health Behavior, Gillings School of Global Public Health, University of North Carolina at Chapel Hill, Chapel Hill, North Carolina, United States of America; 3 Lineberger Comprehensive Cancer Center, University of North Carolina at Chapel Hill, Chapel Hill, North Carolina, United States of America; 4 Hussman School of Journalism and Media, University of North Carolina at Chapel Hill, Chapel Hill, North Carolina, United States of America; 5 Department of Health Promotion, Education, and Behavior, Arnold School of Public Health, University of South Carolina, Columbia, South Carolina, United States of America; 6 Department of Family Medicine, University of North Carolina at Chapel Hill, Chapel Hill, North Carolina, United States of America; University of Saskatchewan, CANADA

## Abstract

**Background:**

Many interventions aim to reduce youth tobacco use, but few have focused on youth who use multiple tobacco products (MTPs). This qualitative study sought to understand how youth who use MTPs view tobacco product risks, describe reasons for MTP use, and perceive barriers and facilitators to cessation.

**Methods:**

We conducted seven virtual focus groups with 30 US youth ages 14–20 years who reported using e-cigarettes and smoking a combustible tobacco product in the past 30 days. We used a semi-structured focus group guide to ask youth about perceived health risks of tobacco products, reasons for MTP use, and thoughts about quitting. We conducted a thematic analysis of transcripts.

**Results:**

The mean age of participants was 18.7 years; 47% identified as white. Most participants were female (63%) and lesbian, gay, or bisexual (63%). Three central themes emerged: 1) uncertainty or misperceptions about e-cigarettes were common, including what ingredients they contain, whether they are tobacco products, and their harm relative to cigarettes; 2) convenience and social factors were mentioned as reasons for using MTPs, rather than using e-cigarettes to quit cigarettes; 3) barriers to cessation included nicotine dependence (especially to e-cigarettes) and environmental factors, while cessation resources were rarely mentioned.

**Conclusions:**

These findings suggest that educational interventions to reduce youth MTP use could focus on correcting misperceptions about e-cigarettes and communicating the harms of combustible tobacco use. Furthermore, behavioral interventions could capitalize on peer and social support while acknowledging unique barriers resulting from MTP use, such as high nicotine dependence.

## Introduction

Tobacco use remains the leading cause of preventable death in the US [1]. In recent years, the tobacco product landscape has become increasingly diverse, leading to the rise of multiple tobacco product (MTP) use, defined as the use of two or more tobacco products in the past 30 days [2]. Among high school-aged youth in the US who reported past 30-day tobacco use in 2024, around one-third used more than one tobacco product [3]. This translates to approximately 580,000 high school-aged youth who use MTPs. Among youth, MTP use is a particularly harmful pattern of tobacco use since it is associated with higher nicotine dependence symptoms [4], decreased intentions to quit tobacco use [4], fewer quit attempts [5], increased risk of problematic substance use [6–8], and decreased academic performance [6] compared with single tobacco product use.

The patterns of MTP use, including which products are used and by whom, are complex. Currently, the most common combination of MTP use among youth involves the use of e-cigarettes with combustible products (i.e., cigarettes or cigars) [9]. However, this may change as new products are added to the marketplace, such as nicotine pouches. In addition, while more data are needed, some groups of people appear to use MTPs at higher rates [10–12]. For instance, among youth, increasing age (i.e., 15 years of age and older) and identifying as male are associated with a higher likelihood of MTP use vs. single tobacco product use [10,11]. Data also suggest potentially higher use of MTPs among youth who identify as lesbian, gay, or bisexual (LGB) [12]. For instance, one study among youth found that LGB males and females had two and four times greater risk of MTP use than straight males and females, respectively [12]. Moreover, research suggests that MTP use may be higher among youth of lower socioeconomic status [13].

Despite vast quantitative research on perceptions of tobacco product risks and patterns of adult MTP use, few qualitative studies have explored risk perceptions and reasons for use among youth who use MTPs [14–19]. Prior qualitative research with young adults shows that those who use MTPs often report lower perceived harm of tobacco products such as cigarettes and e-cigarettes when compared to those who use only one or no tobacco products [14–16]. Social influences, experimentation, and product availability/regulations are also common reasons for MTP use among young adults [15,17–19]. Given the changing landscape of both tobacco product diversity and campaigns about tobacco product risks, up-to-date research is needed to better understand perceptions of health risks and reasons for use among youth who use MTPs.

Other qualitative studies have examined barriers or facilitators of tobacco cessation in youth who use MTPs [15,17,19–23]. While some studies among youth and young adults who use MTPs report that e-cigarettes aid in the cessation of cigarette smoking [15,17,20,21], other studies found that not all youth and young adults who report dual use rely on e-cigarettes to quit smoking cigarettes [15,19]. Beyond the potential role of e-cigarettes in cessation, little is known about factors that impact cessation among youth who use MTPs. One study found that family responsibilities and health concerns may motivate tobacco cessation among young adults who use MTPs [22], but barriers and facilitators to cessation were not thoroughly investigated. Another study among young adults who use MTPs found that social influences, stress, and withdrawal symptoms were barriers to tobacco cessation, with many individuals showing a lack of interest in quitting e-cigarettes [23]. Because MTP use can complicate tobacco cessation, more research is needed on how youth who use MTPs perceive tobacco cessation, including barriers and facilitators.

While many interventions have been implemented to reduce youth tobacco use [24–26], few have focused specifically on MTP use. To inform future behavioral and educational interventions focused on commercial tobacco product use, the goal of this qualitative study was to understand how youth who use MTPs view tobacco product risks, describe reasons for MTP use, and perceive barriers and facilitators to cessation.

## Materials and methods

### Participants

From January 28, 2023 to October 26, 2023, we recruited a convenience sample of participants via ads posted nationally on Instagram and flyers posted locally in the Triangle area of North Carolina. Individuals interested in study participation were prompted to complete an eligibility screener, which assessed participants’ age and place of residence, as well as the type of tobacco products used in the past 30 days, including e-cigarettes, cigarettes, and cigars (i.e., little cigars, cigarillos, and large, traditional cigars). Participants were eligible for study participation if they 1) were between the ages of 13–20 years, 2) reported use of e-cigarettes and a combustible tobacco product (i.e., cigarettes or cigars) within the past 30 days, and 3) lived in the United States (US). Members of the research team contacted eligible participants via phone or email to confirm eligibility, obtain consent, and schedule focus groups. Participants ages 18 years and older provided written consent, and minors who were 17 years or younger provided written assent. We also obtained parental written or verbal consent for minors.

### Procedures

The University of North Carolina at Chapel Hill Institutional Review Board approved the study procedures. Participants were informed that the research team was interested in learning about how to develop tobacco prevention messages for youth. Using an iterative process, the research team developed a focus group guide that included questions about participants’ perceived health risks of different tobacco products, personal tobacco use experiences, reasons for MTP use, and thoughts about quitting tobacco (S1 Appendix). To foster conversations, participants were also polled during the focus groups to answer specific questions, including which tobacco product they used first. The focus group guide was pilot-tested internally prior to study commencement. We conducted seven virtual focus groups using Zoom software between February 2023 and January 2024. We stratified focus groups by age, conducting separate focus groups for 18–20 year olds and 13–17 year olds. Each focus group was recorded. The senior author (SDK), who holds a PhD in health behavior and is trained in qualitative research methods, served as the moderator; two members of the research team (SLP, ID)—graduate students in public health with training in health behavior and epidemiologic methods— alternated serving as the notetaker. The focus groups contained three to six participants and lasted 60–80 minutes. Focus groups were conducted until the point of data saturation. The incentive for participation was a $50 gift card.

### Data analysis

Each focus group was transcribed verbatim and imported into ATLAS.ti (Version 23). We developed an initial codebook using deductive coding, based on topics in the focus group guide that pertained to the following research question: How do youth who use MTPs view tobacco product risks, describe reasons for MTP use, and perceive cessation? Three members of the research team (SAC, SLP, ID) gained familiarity with the codebook and established protocols for coding. Next, coders independently read and coded the same transcript and used an inductive approach to identify codes to add to the codebook [27]. All coders then met to review the coded transcript, resolve coding differences, and add definitions to the codebook for clarification. All coders independently re-coded a second transcript. We calculated inter-rater reliability, specifically Krippendorff’s alpha statistic, as 0.799, indicating reasonable reliability. The coders then dual-coded each of the remaining five transcripts, re-coded the original transcript, and resolved discrepancies during weekly meetings. From the coded data generated, we performed “code and retrieve” analyses to review and sort text excerpts [28], and used analytical tools described by Strauss and Corbin, such as comparative analysis, to better understand the data [27]. Next, using a thematic data analysis approach, we identified significant implicit and explicit ideas, themes, and patterns across the coded excerpts. Through subsequent iterative team discussions, we organized and refined themes and selected illustrative, representative quotes for each theme [29].

## Results

Thirty youth aged 14–20 years (mean: 18.7 years) participated in seven focus groups. Most were female (63%), and about half were white (47%); 63% identified as lesbian, gay, or bisexual (Table 1). Overall, 61% reported e-cigarettes as their first tobacco product, 37% reported cigarettes as their first tobacco product, and 10% did not report which tobacco product they used first. Three central themes emerged: 1) there was uncertainty and misperceptions about e-cigarettes, 2) convenience and social factors were key reasons for MTP use, and 3) barriers to cessation were more commonly reported than facilitators.

**Table 1:** Demographic and tobacco use characteristics among focus group participants, N = 30.

Characteristic	n (%)
Age – mean (SD) (range: 14–20)	18.7 (1.4)
Age group	
13–17 years old	4 (13.3)
18–20 years old	26 (86.7)
Gender	
Female	19 (63.3)
Male	6 (20.0)
Non-binary	4 (13.3)
Prefer not to say	1 (3.3)
Sexual orientation	
Straight	9 (30.0)
Lesbian, gay or bisexual (LGB)	19 (63.3)
Other sexual orientation ^a^	2 (6.7)
Socioeconomic status	
Lower	13 (43.3)
Medium	14 (46.7)
Higher	3 (10.0)
Race	
White	14 (46.7)
Black or African American	5 (16.7)
Asian	6 (20.0)
Middle Eastern or Northern African	1 (3.3)
More than one race	3 (10.0)
Other race	1 (3.3)
Hispanic/Latino	3 (10.0)
Past 30-day tobacco use	
E-cigarettes	30 (100.0)
Cigarettes	27 (90.0)
Cigarillos	8 (26.7)
Little cigars	6 (20.0)
Large cigars	7 (23.3)
Past 30-day use frequency ^b^	
E-cigarettes, mean (SD)	12.6 (10.4)
Cigarettes, mean (SD)	9.9 (10.2)
Cigarillos, mean (SD)	4.9 (6.8)
Little cigars, mean (SD)	6 (7.3)
Large cigars, mean (SD)	3 (3.3)

^a^Includes queer (n = 1) and pansexual (n = 1) participants.

^b^Refers to mean number of days product was used among those reporting past 30-day use.

### Theme 1: Youth expressed uncertainty or misperceptions about e-cigarettes, including their ingredients (beyond nicotine), whether they are tobacco products, and their harm relative to cigarettes

Though youth were able to identify nicotine as a common ingredient in e-cigarettes, many were unsure about other ingredients present and whether e-cigarettes were tobacco products. Participants discussed that many e-cigarettes do not list ingredients, “*there’s no like supplement label of the ingredients…*” (19yrs, gender not reported) and because of this, they were unsure about what the products contain, “*There’s so many vapes out there, they have all these different flavors. You don’t really know what they’re putting in this.”* (20yrs, male). Relatedly, the perception that e-cigarettes were not tobacco products was also common, with several factors complicating how youth perceived e-cigarettes, including how some are nicotine-free, *“I wasn’t sure because there’s products that have nicotine and then there’s some that don’t have nicotine in them*.” (14yrs, male), and how they can be made from synthetic nicotine, “…*I know that nicotine comes from tobacco, but most vapes now are synthetic nicotine.”* (19yrs, female). Alternatively, a few participants did classify e-cigarettes as tobacco products, noting that e-cigarettes elicit the same sensation as cigarettes: *“I’m assuming that they are the same and are a tobacco product because they do give you the same effect …”* (20yrs, female).

When comparing the harm of e-cigarettes to cigarettes, the vast majority of participants were either unsure which was more harmful or incorrectly believed that e-cigarettes were equally or more harmful than cigarettes. For those expressing uncertainty, they cited a lack of research on e-cigarettes, “*I feel like just because [e-cigarettes] aren’t really researched and there are so many different types, it’s really hard to say whether vaping in general is worse than cigarettes*…” (20yrs, female), uncertainty about long-term effects, “*with vaping, it just hasn’t been around long enough for people to know how it affects you long term”* (18yrs, female), and mixed information from individuals and the media, *“There’s so many people saying these different things based on their own experiences so it’s hard to get a definite answer.”* (16yrs, female) and *“… e-cigarettes are still being advertised to this day as the healthier alternative to smoking cigarettes. And I’m not a scientist, don’t know if that’s true or not.”* (19yrs, gender not reported).

Participants who thought e-cigarettes were more harmful than cigarettes expressed that this is because e-cigarettes were less “natural”. Specifically, some participants voiced that cigarettes were made from natural substances, *“I would argue vaping is worse ‘cause at least we know how cigarettes are made and that they’re made from natural products while vaping could be anything and we have no idea, and there’s no way to find out.”* (19yrs, gender not reported). The misperception that cigarettes are healthier because they are derived from a plant was persistent, with participants associating e-cigarettes more with chemicals, “*I think that vaping is more harmful than smoking a normal cigarette because tobacco is a naturally grown product and vapes have a bunch of chemicals in them*.” (14yrs, male). Finally, some participants expressed that because e-cigarettes could be used more discreetly and frequently, which increases their addictiveness, they were more harmful than cigarettes, *“I think with the vapes you can use a lot more... you’re able to hide it more. You can use it basically constantly...”* (19yrs, female) or equally harmful as cigarettes because *“…as far as addiction goes, they’re probably about the same.”* (20yrs, non-binary).

Only a few participants thought e-cigarettes were less harmful than cigarettes. One participant mentioned being taught from a young age that cigarettes are harmful, *“I feel like we grew up just engraved in our brain that cigarettes are the worst, so we automatically think vaping’s better.”* (20yrs, female), and a few participants mentioned the availability of more research on cigarette harms,*“…when comparing [e-cigarettes] to cigarettes in my mind, it’s like okay, well one has been proven to kill people and one hasn’t yet.”* (18yrs, female). Participants did not explicitly discuss the risks of MTP use compared to the risks of single tobacco product use.

### Theme 2: Youth frequently described using MTPs for convenience and social reasons, rather than using e-cigarettes to quit cigarettes, which they understood to be a reason why adults use MTPs

Convenience and accessibility were commonly mentioned as reasons for MTP use among participants. Youth frequently discussed how the products they used were based on what was available around them: *“… if you’re at a party and somebody’s got cigarettes, you’re gonna smoke cigarettes. And if you’re at a party and somebody’s got a vape, you’re gonna hit the vape. Or it’s whatever’s cheaper or whatever you found nearby…”* (19yrs, gender not reported). Participants also stated that their environment plays a role in MTP use, noting that e-cigarettes are easier to conceal than cigarettes: *“I know I started with cigarettes, but then got into vapes out of the convenience of being able to hide it.”* (19yrs, male) and *“So I’m in a dorm right now, and I can’t really pull out a cigarette because it’ll alarm the smoke alarms, it’ll smell really strong, it’ll bother my roommates. But a vape, they won’t even notice. But again at a party…I can pull out a cigarette and smoke without bothering anyone really.”* (19yrs, female). Several participants also stated they enjoyed the convenience of alternating between a variety of tobacco products, “*I guess the convenience of whichever is most readily available to them because some people I know also have vapes as well as cigarettes themselves so if their vape ends up dying and they need to buy a new one, they have a backup*.” (20yrs, female). When one participant described which products they used, they stated, “*I think it’s what was available. So sometimes you’ll have a vape in hand and then you’ll lose it and then you go to a party and they have cigarettes so you’ll smoke. I mean that’s what I think*.” (19yrs, female).

Youth also acknowledged different social factors as reasons for MTP use, with many participants describing use of MTPs in social situations to blend in with their friends: “…*if I’m hanging out with this group of friends who I know smoke cigarettes and doesn’t vape, I’m gonna do what they’re doing and vice versa.”* (19yrs, gender not reported) and “*I mean, a lot of it is pressure. Just if you’re in a social situation, your friends will be vaping or might be smoking a cigarette and you want to join in.”* (20yrs, non-binary).

While discussing methods to quit tobacco products, youth acknowledged that e-cigarettes could be used to quit cigarettes, but this was seen as something only adults did and not applicable to youth: *“I definitely think that’s more for older people. A lot of people I know, I, myself included, started vaping before they tried cigarettes.*” (20yrs, female). Because many participants started using e-cigarettes first, they expressed that switching to e-cigarettes was not a viable cessation method: *“I think generally speaking…it [e-cigarette] is really not used as much to quit smoking cigarettes for Gen Z and younger people … I think that people… go straight to vape as opposed to cigarettes.”* (18yrs, female) and *“… nobody that I know who uses nicotine products has used vaping to quit cigarettes”* (19yrs, male). Instead, to quit using e-cigarettes, one participant switched to cigarettes because they viewed cigarettes as having less nicotine: “*But when I was quitting, I used cigarettes to help me quit ‘cause it made me like grossed out by nicotine. And it’s also so much less nicotine than a vape.”* (20yrs, female).

### Theme 3: Youth frequently discussed barriers to cessation, including nicotine dependence (especially to e-cigarettes) and environmental factors; however, cessation resources were rarely mentioned

When asked about barriers to cessation, youth frequently discussed their nicotine dependence or addiction, and many noted that they were especially addicted to e-cigarettes compared to cigarettes: *“The nicotine feels different too. Like vaping can feel more strong than cigarettes and the rush is stronger.”* (19yrs, non-binary). Some participants lamented that because they started using e-cigarettes first, their tolerance for nicotine was higher: *“Like a vape is like taking a shot and a cigarette is like drinking a beer, and if you’re 14 and you start taking shots, a beer’s not gonna do anything for you ‘cause you’ve already like become tolerant I guess you know.”* (19yrs, gender not reported). Many expressed an inability to quit or lack of control given the strong withdrawal and resulting relapse they experienced: “*I think about withdrawal symptoms sometimes. Sometimes you might want to stop, but they still feel the urge to smoke and then that’s more powerful than your urge to quit so you continue smoking.”* (17yrs, female) and *“… relapsing is really real and it’s really hard too - ‘cause you’re then battling with a lot of guilt. There’s a lot of guilt around nicotine addiction...”* (20yrs, female).

Some participants indicated that they had tried to quit tobacco products; however, many participants expressed that they did not have social support. For example, one participant discussed how they felt adults do not know how to deal with addiction: *“I think like adults and like family figures aren’t really at least in my experience well versed in how to deal with somebody who is trying to quit…it is still an addiction…and I don’t think people are very like educated in dealing with that.”* (19yrs, gender not reported). Participants also voiced how it is hard to quit because tobacco products are easily accessible. This included being around peers, *“… if I’m hanging out with my friends and they’re all outside smoking cigarettes, then it’s way harder to turn a cigarette down if it’s offered to you because it’s an easy way to socialize honestly especially if you’re out.”* (20yrs, female) or partaking in usual daily activities, *“Even if you’re not at a party, you’re just walking down the road, you’ll see people hitting their vapes. It’s just common everywhere so it’s really hard to escape it.”* (19yrs, female).

One participant mentioned how the placement of smoke shops and the lack of age verification from store clerks in their neighborhood make it easy to obtain and continue using tobacco products: *“…it’s extremely easy to get. There’s a smoke shop on every other block, multiple even, and they’re never gonna ask for ID. It’s very quick and easy to get when you can just go in and go out in less than thirty seconds and you can get one right away.”* (19yrs, female). Another participant commented on the availability of tobacco products, specifically e-cigarettes, online: *“In my opinion, getting vapes would be easier than getting cigarettes especially since I’ve also seen on Instagram and TikTok of random sketchy accounts that are advertising selling them online for people who are under 21.”* (20yrs, female).

While many acknowledged a lack of social support and product availability as barriers to cessation, few acknowledge resources or facilitators to cessation. When resources were mentioned, participants often described how peers could provide support and encouragement to help them quit tobacco products: *“In my experience, when I’ve had friends that have tried to quit, or like half-quit vaping or smoking, it’s easier because of talking to other people who have, versus consulting the internet.”* (19yrs, female). Many participants also recognized that separating themselves from peers who use tobacco can help with quitting: “*I think one of the most important things would be the type of people we surround themselves with. Obviously if you hang around people who smoke a lot, you’re gonna be bound to smoke if you’re used to smoking. And if you hang around people who are not really smokers, then you won’t be urged to smoke”* (17yrs, female). For others, decreasing product accessibility in the community was seen as a way to help youth quit. One method suggested was for stores to confirm proof of age when selling tobacco products: *“I think especially if someone’s under 21, the biggest thing that can help is getting it out of their reach. I know in periods where I’ve stopped, a lot of it has just been because of access. If my friends don’t have vapes or there’s not a store nearby that I know doesn’t ID, then I literally can’t get them so I think making it less accessible in that way.”* (19yrs, male).

## Discussion

This qualitative research explored how youth who use MTPs view tobacco product risks, their reasons for MTP use, and how they perceive barriers and facilitators to cessation. Findings revealed great uncertainty and misperceptions surrounding e-cigarettes, which is important because misperceptions are associated with tobacco use [30], and may contribute to MTP use. We found that youth could clearly identify nicotine as an ingredient in e-cigarettes but were unsure about other ingredients or chemicals, which is consistent with prior studies [30,31]. There is clear messaging in the US that e-cigarettes contain nicotine through prevention campaigns that often focus on nicotine or nicotine addiction [32–34]. While many campaigns also discuss other chemicals found in e-cigarettes, youth in our study were overwhelmingly unsure about what else e-cigarettes contained. This is potentially due to the wide diversity of e-cigarette products available, including many products that have not been authorized for sale in the US (i.e., flavored disposable e-cigarettes) and are on store shelves illegally [35,36]. Relatedly, participants noted confusion about whether e-cigarettes could be classified as “tobacco products,” in part due to the availability of nicotine-free and synthetic nicotine e-cigarette products. Given the advent of new products like synthetic nicotine and nicotine-free e-cigarettes, and the use of e-cigarettes to vape other substances (e.g., THC), public health education about the harms of nicotine (whether it is synthetic or not), nicotine-free e-cigarettes, and tobacco and cannabis co-use (e.g., campaigns about vaping THC) may be warranted.

Relatedly, the majority of youth in our study were unsure about the relative harm of e-cigarettes compared to cigarettes or believed that e-cigarettes were more harmful than cigarettes. Though prior studies show that many adolescents feel that e-cigarettes are safer or less harmful than combustible cigarettes [30,31,37,38], we found the reverse—many participants in our study thought e-cigarettes were equally or more harmful than cigarettes. Concerningly, some youth even perceived cigarettes to be more “natural” than e-cigarettes because cigarettes contain natural tobacco leaf, whereas e-cigarettes are made in labs. In response to growing e-cigarette use in recent years, most tobacco prevention campaigns targeting youth have shifted towards focusing on e-cigarettes [39]. While these campaigns are essential, efforts to communicate about the harms of combustible tobacco use, including cigarettes and cigars, are still needed. Indeed, some youth in our study discussed trying to quit e-cigarettes by switching to cigarettes. While no level of tobacco use is safe in youth, efforts to provide clear messaging regarding the absolute and relative harm of tobacco products to youth who use MTPs should be developed and evaluated.

Consistent with prior research [14,18], youth in our study often spoke about using MTPs for convenience, accessibility, and social reasons, not smoking cessation. This contrasts with adults who cite quitting smoking as a common reason for MTP use [40,41]. Specifically, youth mentioned using different tobacco products depending on social situations or environmental factors, like being able to conceal e-cigarettes in places where cigarettes could not be used. Therefore, adopting tobacco-free policies that include all nicotine products in school and on college campuses can be beneficial to reducing MTP use among youth but does not address all reasons for MTP use, including the concealability of e-cigarettes and the ability to “stealth vape” [42]. The ability to easily purchase different tobacco products from online retailers and smoke shops was also cited as a reason for MTP use among participants. Though raising the minimum age of sale of tobacco products reduces tobacco use among youth [43,44], effective enforcement is needed to significantly impact youth tobacco use [45]. In 2020, after the enactment of the federal Tobacco 21 legislation, only 17% of middle and high school students in the US were unsuccessful at buying tobacco products due to their age [24]. Thus, policies and programs to promote age verification are needed to reduce youth access to tobacco products, including thorough compliance checks at tobacco retailers.

We also found that youth frequently described nicotine dependence, especially to e-cigarettes, and mentioned starting e-cigarettes before combustible products [46]. This trend appears to contrast with trajectories of MTP use among adults who have historically smoked cigarettes first and then used e-cigarettes to aid in cessation [47–51]. The use of e-cigarettes in the absence of combustible cigarettes has been found to increase the risk of smoking cigarettes among youth and young adults [52,53], likely contributing to MTP use among this age group. As many participants expressed starting out with e-cigarettes, more stringent policies surrounding e-cigarette accessibility for youth may aid in reducing MTP use in the future. Because many participants started using e-cigarettes first, some also expressed they did not have a way to quit using tobacco as easily as adults who use e-cigarettes as a cessation aid. These findings align with prior studies suggesting that e-cigarettes may be contributing to unsuccessful nicotine quit attempts [54], particularly among adolescents [55,56], and that nicotine concentrations have increased in recent years.

Importantly, youth were able to identify multiple barriers to cessation but were less able to identify evidence-based resources. This is consistent with research showing that effective cessation resources for youth who use tobacco products are scarce [57]. Moreover, most cessation resources were created for cigarette smoking cessation and do not directly address specific products highly relevant to youth, such as e-cigarettes. Although there is overlap in barriers to cessation for both vaping and smoking, there are also unique differences between the two, such as the wide variety of e-cigarette flavors and the ability to use e-cigarettes discreetly [58]. In addition, many cessation resources were created for cessation of single tobacco products rather than MTPs [59]. As such, there remains a need for cessation interventions and resources that are directly relevant to youth who use MTPs and address their barriers to cessation. While interventions addressing MTP use are needed, they should not come at the expense of preventive interventions for youth who do not use any tobacco products, which remain vital to preventing future tobacco product use among youth.

Finally, focus group participants also mentioned the benefit of social influences in cessation, suggesting that future interventions should consider incorporating peer and social support to aid in cessation for MTPs. A systematic review of smoking cessation interventions for young adults found that promising interventions included components focused on peer support [59]. Our findings, in concert with previous research, suggest that interventions addressing youth MTP could incorporate peer support, but more research is needed.

### Strengths and limitations

Strengths of our study include our focus on a high priority population for tobacco cessation interventions—youth who use MTPs; our use of multiple checks to ensure we recruited actual youth who use MTPs, including verification of all screening criteria in zoom calls before focus groups were scheduled; and the diversity of participants by race, SES, and sexual orientation. Indeed, while our study did not intentionally recruit LGB youth, almost two-thirds of our sample reported being LGB. This is perhaps because 1) more youth identify as LGB compared to adults; 2) MTP use appears to be higher among LGB youth and young adults [12,60,61]; and 3) we recruited via social media, and LGB youth use social media at higher rates than straight youth [62]. Future research could examine reasons for MTP use specifically among LGB youth.

Our findings should also be interpreted with limitations in mind. Our recruitment window was relatively long due to difficulties reaching our population and enrolling eligible participants. In addition, as this was a convenience sample with only 30 participants, results may not be generalizable to youth across the US who use MTPs. Moreover, this study focused exclusively on past 30-day use of e-cigarettes and cigarettes/cigars, which does not reflect other types of MTP use (e.g., all non-combustible use or all combustible use). Research examining the experiences and perspectives of individuals reporting other types of MTP use is needed. Finally, while we aimed to examine perceptions and experiences among youth ages 13–20, we only conducted one focus group with minors under the age of 18, which limits the generalizability of findings to younger-aged adolescents who use MTPs. However, it is important to note that MTP use is more common among older adolescents [10,11], which is reflected in our sample. Relatedly, our original goal was to analyze data separately for 13–17 year olds vs. 18–20 year olds. However, because we were only able to conduct one focus group with 13–17 year olds, we were not able to explore differences by age group.

### Conclusions

Findings from our qualitative study suggest that educational interventions to reduce youth MTP use could focus on correcting misperceptions about e-cigarettes and communicating about the harms of combustible tobacco use. In addition, behavioral interventions could capitalize on peer and social support while acknowledging unique barriers resulting from MTP use, such as high nicotine dependence.

## Supporting information

S1 Appendix Focus group guide. (DOCX)

S2 Appendix COREQ checklist. The consolidated criteria for reporting qualitative research (COREQ): 32-item checklist.
(DOCX)
