# Supplementary material for: Perceived risks, reasons for use, and barriers to cessation among youth who use multiple tobacco products
Source: PLoS One. 2025 Nov 7;20(11):e0335019. doi: 10.1371/journal.pone.0335019 (PMC12594371; doi:10.1371/journal.pone.0335019)
Supplement: S1 Appendix — (DOCX) [file pone.0335019.s001.docx]

**S1 Appendix. Focus Group Guide**

**Welcome**

Welcome and thank you for joining today. We are conducting focus groups to learn more about how adolescents and young adults perceive tobacco products. We appreciate your time today because your insights are very important to us. Our discussion will last about an hour and 30 minutes.

**Topic: Personal tobacco use experiences**

1. First, we want to hear more about your personal experiences with tobacco. We would like to do a quick poll. These poll questions are anonymous. We won’t know who says what.

| **Zoom Poll:**  What tobacco product did you use first?   1. E-cigarettes or vapes 2. Cigarettes 3. Cigars 4. Smokeless tobacco 5. Hookah 6. Some other product not listed |
| --- |

Okay, so it looks like… [Comment on summary of poll].

**Topic: Reasons for tobacco use**

1. We know that people your age use tobacco products such as vapes, cigarettes, and cigars for lots of different reasons. Please share some reasons why you think people your age use vapes and other tobacco products?

**Probes**: Flavors? Curiosity? Peers? Enjoyment? Relieve stress or anxiety? Boredom? Do any of those reasons ring true for you and why or why not?

1. We would like to hear from the group reasons why you think people your age use more than one tobacco product?

**Probes**: Displacement (i.e., vaping to reduce how much you smoke?) Experimentation (i.e., became curious about another product?) Instrumentality or context (i.e., different products can be used in different environments or situations?)

**Topic: Perceived health risks**

1. Now, we would like to hear more on your thoughts on tobacco products using another zoom poll.

| **Zoom Poll**  Do you think vapes or e-cigarettes are a tobacco product?   1. Yes 2. No 3. Not sure   How harmful do you think vapes or e-cigarettes are?   1. Not harmful 2. Somewhat harmful 3. Very harmful |
| --- |

Let’s talk about the first question. For people who said “no”, could you say more about that? For people who said “yes”, could you say more about that? And what about people who said “not sure”?

Now let’s talk about the second question. For people who said “not harmful,” could you say more about that? For people who said “somewhat harmful” or “very harmful,” could you say more about that?

1. How do you think the health risks of vaping and smoking cigarettes compare?

**Probe**: Do you think vaping is more or less harmful than smoking cigarettes? Why or why not? Do you think vaping is more or less harmful than smoking cigars? Why or why not?

**Topic: Quitting**

1. Quitting tobacco products is difficult for many people. What do you think helps young people quit vaping or quit smoking?

**Probe**: Are there any resources that you could think of that would be helpful? Is there any information or facts that could help young people to quit vaping and smoking? Medications? Free? Coach? An incentive? Do you think young people would prefer to quit one product first and then quit another or quit both at the same time and why?

**Probe**: For specific resources mentioned, how would those help you to quit?

1. What do you think are some challenges that young people face when they try to quit vaping or quit smoking?

**Probe**: Stress? Lack of information on risks or strategies to quit? Social factors? Addictiveness of products?

**Topic: Closing**

This conversation has been very helpful. Do you have any questions for me or final thoughts? Is there anything important related to addressing vaping or smoking cigarettes or cigars that we missed?

Thank you again for participating today.
