# Supplementary material for: Perceived risks, reasons for use, and barriers to cessation among youth who use multiple tobacco products
Source: PLoS One. 2025 Nov 7;20(11):e0335019. doi: 10.1371/journal.pone.0335019 (PMC12594371; doi:10.1371/journal.pone.0335019)
Supplement: S2 Appendix — The consolidated criteria for reporting qualitative research (COREQ): 32-item checklist. (DOCX) [file pone.0335019.s002.docx]

**S2 Appendix. COREQ Checklist.** The Consolidated Criteria for Reporting Qualitative Research (COREQ): 32-item Checklist

| Item | Guide questions/description | Notes |
| --- | --- | --- |
| **Domain 1: Research Team and Reflexivity** | | |
| Personal Characteristics |  |  |
| 1. Interviewer/facilitator | Which author/s conducted the interview or focus group? | SDK conducted the focus groups. SLP and ID served as notetakers during the focus groups.  Described in methods. |
| 1. Credentials | What were the researcher’s credentials? | SDK: PhD  SLP: MPH  ID: BS  Described in methods. |
| 1. Occupation | What was their occupation at the time of the study? | SDK: Assistant Professor  SLP: Graduate research assistant  ID: Graduate research assistant  Described in methods. |
| 1. Gender | Was the researcher male or female? | SDK, SLP, and ID were females. |
| 1. Experience and training | What experience or training did the researcher have? | SDK, SLP, and ID had experience with facilitating focus groups and conducting participant interviews.  Described in methods. |
| Relationship with Participants |  |  |
| 1. Relationship established | Was a relationship established prior to study commencement? | The research team had no prior contact with participants prior to study commencement. |
| 1. Participant knowledge of the interviewer | What did the participants know about the researcher? | Participants were informed that the research team was interested in learning about how to develop tobacco prevention messages for youth.  Described in methods. |
| 1. Interviewer characteristics | What characteristics were reported about the interviewer/facilitator? | Participants were told that the moderator and note-takers worked at UNC Chapel Hill. |
| **Domain 2: Study Design** | | |
| Theoretical Framework |  |  |
| 1. Methodological orientation and theory | What methodological orientation was stated to underpin the study? | The research team used a thematic data analysis approach. We analyzed the data using both deductive (developed an initial codebook based on topics in the focus group guide that pertained to the research question) and inductive (identified codes to add to the codebook after reading and coding transcripts) codes.  Described in methods. |
| Participant Selection |  |  |
| 1. Sampling | How were participants selected? | Participants were recruited using convenience sampling.  Described in methods. |
| 1. Method of approach | How were participants approached? | Participants were recruited via ads posted nationally on Instagram and flyers within the Triangle area of North Carolina. Individuals interested in study participation were prompted to complete an online eligibility screener and eligible participants were contacted via phone or email to confirm eligibility, obtain consent, and schedule focus groups.  Described in methods. |
| 1. Sample size | How many participants were in the study? | 30 participants were included in the study.  Described in results. |
| 1. Non-participation | How many people refused to participate or dropped out? | No people refused to participate or dropped out after beginning participation. |
| Setting |  |  |
| 1. Setting of data collection | Where was the data collected? | Focus groups were conducted virtually using Zoom software.  Described in methods. |
| 1. Presence of non-participants | Was anyone else present besides the participants and researchers? | There was no one else present aside from the participants and researchers. |
| 1. Description of sample | What are the important characteristics of the sample? | The mean age of participants was 18.7 years; 47% identified as white. Most participants were female (63%) or lesbian, gay, or bisexual (63%).  Described in results. |
| Data Collection |  |  |
| 1. Interview guide | Were questions, prompts, guides provided by the authors? Was it pilot tested? | The focus group guide was provided as a supplemental document (See S1). The focus group guide was pilot tested internally prior to study commencement.  Described in methods. |
| 1. Repeat interviews | Were repeat interviews carried out? If yes, how many? | There were no repeat interviews carried out with participants. |
| 1. Audio/visual recording | Did the research use audio or visual recording to collect the data? | All interviews were audio recorded with consent from all participants.  Described in methods. |
| 1. Field notes | Were field notes made during and/or after the interview or focus group? | The focus group notetakers (SLP, ID) made field notes during the focus groups. |
| 1. Duration | What was the duration of the interviews or focus group? | Focus groups lasted 60-80 minutes on average.  Described in methods. |
| 1. Data saturation | Was data saturation discussed? | Data saturation was reached for all major topics.  Described in methods. |
| 1. Transcripts returned | Were transcripts returned to participants for comment and/or correction? | Transcripts were not returned to participants. |
| **Domain 3: Analysis and Findings** | | |
| Data Analysis |  |  |
| 1. Number of data coders | How many data coders coded the data? | Three members of the research team (SAC, SLP, ID) coded the data.  Described in methods. |
| 1. Description of the coding tree | Did authors provide a description of the coding tree? | A description of the codebook and coding process are described in the methods. |
| 1. Derivation of themes | Were themes identified in advance or derived from the data? | Themes were derived from the data.  Described in methods. |
| 1. Software | What software, if applicable, was used to manage the data? | All transcripts were coded using Atlas.ti software.  Described in methods. |
| 1. Participant checking | Did participants provide feedback on the findings? | Participants did not provide feedback on the findings. |
| Reporting |  |  |
| 1. Quotations presented | Were participant quotations presented to illustrate the themes / findings? Was each quotation identified? | Quotations and participant identifiers (age and gender) were included in the results of the manuscript.  Described in results. |
| 1. Data and findings consistent | Was there consistency between the data presented and the findings? | All findings emerged directly from the data, and each theme is supported with participant quotes.  Described in methods and results. |
| 1. Clarity of major themes | Were major themes clearly presented in the findings? | Major themes were presented in the results along with supporting quotations.  Described in results. |
| 1. Clarity of minor themes | Is there a description of diverse cases or discussion of minor themes? | Themes that included opinions mentioned by only a single or few participants are addressed in the results section.  Described in results. |
